# In Regards to Hayashi et al. JACMP 27(8), e70741

**DOI:** 10.1002/acm2.70827

**Published:** 2026-10-09

**Authors:** Jicheng Zhang, Tao You

**Affiliations:** ^1^ Department of Radiation Oncology Affiliated Hospital of Jiangsu University Zhenjiang Jiangsu China

1

In Regard to Hayashi et al.

We read the study by Hayashi et al.^1^ with interest. The authors evaluated how MRI slice thickness and gold fiducial marker (GFM) orientation affect measurement accuracy in three‐dimensional multi‐echo gradient recalled echo imaging. They found lower measurement error with 1.0‐mm slices and concluded that this protocol can maintain “consistent sub‐millimeter localization accuracy” across marker orientations. The study provides useful data on marker visualization, but the reported endpoint does not directly measure localization error.

The primary outcome was the difference between the GFM length calculated on the treatment planning system and its physical length of 10.0 mm.^1^ Observers identified the three‐dimensional coordinates of both marker tips, but these coordinates were then used to calculate the Euclidean distance between the tips. Thus, the reported error is an error in measured marker length, not the displacement of either tip or of the marker relative to an independent spatial reference. If both identified tips are shifted by the same vector, the measured length can remain unchanged despite a localization error. Conversely, the apparent signal void could extend at both marker ends, increasing the measured length while leaving the marker center unchanged. The authors themselves note in the Discussion that stability in length measurement does not imply true three‐dimensional localization accuracy because their metric does not measure coordinate deviation relative to a reference.^1^ This distinction is not carried into the Conclusion.

The data also do not show submillimeter marker‐length error at every orientation. With 1.0‐mm slices, the overall mean measurement error was 0.96 mm (95% CI, 0.70‐1.22 mm).^1^ In Table 3, the orientation‐specific means range from 0.32 to 1.42 mm, and 7 of the 12 orientations exceed 1 mm.^1^ These results support lower overall marker‐length error with 1.0‐mm slices, but not the statement that submillimeter accuracy was maintained across all orientations.

This also affects the margin illustration. The authors use the maximum 3.10‐mm measurement error as a hypothetical constant systematic shift in the Van Herk margin formula.^1^, ^2^ A 3.10‐mm overestimation of marker length, however, is not a demonstrated 3.10‐mm translational displacement. For example, extension of the signal void at both marker ends could increase the measured length while leaving the marker center unchanged. The authors appropriately describe this calculation as theoretical, but treating a length error as an equivalent positional error goes beyond what the experiment measured.

The advantage of thinner slices remains supported by the study. We suggest describing that advantage in terms of reduced marker‐length measurement error unless localization is assessed against independently known spatial reference coordinates. Direct comparison of marker‐tip or centroid coordinates with an independent spatial reference would assess localization more directly. Registration accuracy relevant to CT‐MRI fusion could then be evaluated using established image‐registration frameworks.^3^ For the present study, whether the 1.0‐mm protocol also provides submillimeter CT‐MRI localization remains to be demonstrated.

## CONFLICT OF INTEREST STATEMENT

The authors declare that the research was conducted in the absence of any commercial or financial relationships that could be construed as a potential conflicts of interest.

## AI GENERATIVE STATEMENT

During the preparation of this manuscript, the authors used ChatGPT to assist with language editing. The authors independently verified the scientific content, numerical data, and references, reviewed and revised the final manuscript, and take full responsibility for its content.

## References

[1] Hayashi A , Takahashi Y , Sanuki N , et al. Impact of MRI slice thickness and fiducial marker orientation on geometric accuracy in multi‐echo GRE imaging for prostate radiotherapy planning. J Appl Clin Med Phys. 2026;27:e70741. 10.1002/acm2.70741 PMC13473699 42596050

[2] van Herk M , Remeijer P , Rasch C , Lebesque JV . The probability of correct target dosage: dose‐population histograms for deriving treatment margins in radiotherapy. Int J Radiat Oncol Biol Phys. 2000;47(4):1121‐1135. 10.1016/S0360-3016(00)00518-6 10863086

[3] Brock KK , Mutic S , McNutt TR , Li H , Kessler ML . Use of image registration and fusion algorithms and techniques in radiotherapy: report of the AAPM radiation therapy committee task group no. 132. Med Phys. 2017;44(7):e43‐e76. 10.1002/mp.12256 28376237

